# Social norm change: drivers and consequences

**DOI:** 10.1098/rstb.2023.0023

**Published:** 2024-03-11

**Authors:** Giulia Andrighetto, Sergey Gavrilets, Michele Gelfand, Ruth Mace, Eva Vriens

**Affiliations:** ^1^ Institute of Cognitive Sciences and Technologies, Italian National Research Council, Rome 00185, Italy; ^2^ Department of Ecology & Evolutionary Biology, Department of Mathematics, University of Tennessee, Knoxville, TN 37996-1610, USA; ^3^ Graduate School of Business and Department of Psychology, Stanford University, Stanford, CA 94305, USA; ^4^ Department of Anthropology, University College London, London WC1H 0BW, UK

**Keywords:** social norms, norm change, evolution, culture, intervention

## Abstract

Social norms research is booming. In recent years, several experts have recommended using social norms (unwritten rules that prescribe what people ought or ought not to do) to confront the societal, environmental and health challenges our societies face. If we are to do so, a better understanding is required of how social norms themselves emerge, evolve and respond to these challenges. Social norms have long been used as *post hoc* explanations of behaviour or are seen as stable social constructs. Yet norms evolve dynamically with the changing group processes (e.g. political polarization, kinship structures) and societal challenges (e.g. pandemics, climate change) for which they are presented as solutions. The Theme Issue ‘Social norm change: drivers and consequences' contains 14 contributions that present state-of-the-art approaches to understand what generates social norm change and how this impacts our societies. Contributions give insight into (i) the identification of norms, norm change and their effect on behaviour; (ii) drivers and consequences of spontaneous norm change; and (iii) how norm change can be engineered to promote desired behavioural change.

This article is part of the theme issue ‘Social norm change: drivers and consequences’.

## 1. Introduction

Social norms are unwritten rules that are collectively understood, prescribe what people ought (not) to do, and motivate people to engage in individually costly but socially beneficial behaviour [1–3]. In recent years, scientists and experts have appealed to resort to social norms to confront the societal, environmental and health challenges that our societies face [4–7]. Indeed, social norms have contributed in a crucial way to, for example, the reduction of smoking in public places [8], the increase in eco-friendly behaviour [9–12] and the decrease of COVID-19 cases and deaths [6,13]. If we are to truly leverage social norms, however, we need to better understand how they emerge, evolve and respond to changes.

Most research on social norms has abstracted away from their dynamic nature. The number of studies using norms and norm-based interventions to generate behavioural change has grown exponentially, but many are conducted without knowledge of the norm in place. Without explicit measurement of norms or their effect on behaviour, norms have been interpreted as stable social constructs and used as *post hoc* explanations of observed behavioural patterns. Most studies focus on causal effects of (already established) norms on behaviour or use norm nudges and interventions to strengthen their effect. Yet whether or not interventions work and how remain a black box unless underlying norm dynamics are understood. Norms evolve dynamically with the changing group processes (e.g. political polarization, kinship structures) and societal challenges (e.g. pandemics, climate change) for which they are presented as collective solutions. Once we recognize that social norms themselves may change in response to the challenges they are targeted to solve, their effectiveness in guiding (socially desirable) behaviour is far from guaranteed [14]. Understanding the evolution of social norms—how and why they emerge, spread, change or remain stable—is crucial to develop norm-based interventions benefiting society and its individual members (for a recent overview see [15]).

New perspectives and theories are needed to map the evolution of social norms, disentangle mechanisms driving change and derive consequences of these changes. This Theme Issue integrates and advances knowledge on norm change with contributions that place norms at the centre, rather than using them as (abstract) tools to explain behaviour. Our goal with this Theme Issue is to present and integrate state-of-the-art research and methodologies across disciplines. We put together some of the most promising developments from various disciplinary backgrounds and methodological approaches, including cultural and social psychology, evolutionary biology, cognitive sciences, anthropology, complex systems, economics and sociology, and covering different methods, including behavioural experiments, surveys, ethnographic and historical research, and computational and simulation models, in the study of social norms *evolution and change*. Contributions give insight into (i) the identification of norms, norm change and their effect on behaviour; (ii) drivers and consequences of spontaneous norm change; and (iii) how norm change can be engineered to promote desired behavioural change. Anticipating a boost in social norms research, this Theme Issue could serve as a cornerstone for theoretical and conceptual convergence across disciplines, allowing the involved fields to advance and sustainable policy interventions to be identified.

## 2. Overview of this theme issue

### (a) Identification of norms, norm change and their effect on behaviour

The first section introduces the concept of social norms and how they are identified and diagnosed. Despite some recent advances [16–19], there still is a substantial ambiguity in how social norms are inferred. The contributions of this Theme Issue identify social norms both in humans and in animals and discuss models of norms dynamics that help understanding of how norms emerge, persist and change in a variety of settings, both controlled and uncontrolled. This section includes a review article on modelling approaches to understand norm change [20], an opinion article on the evolution of normativity in non-human animals [21], a research article on the impact of normative expressions on norm perception and compliance [22], and a research article on the inference of social norms from policy signals [23].

Gavrilets *et al*. [20] review existing theoretical approaches for modelling the origin, persistence and change of social norms. The authors present an integrative approach that combines norm-utility approaches with beliefs dynamics and examine how this novel approach makes it possible to effectively model the emergence, persistence and evolution of social norms. They also discuss future directions in the modelling of social norms, in particular the need of incorporating network structure more thoroughly, studying online norms, considering cultural variations and the application of insights from modelling for the mitigation of various challenges faced by our societies.

Challenging standard positions in the literature, Andrews *et al*. [21] propose that social norms are not unique to humans but are also present in animal cultures. The authors argue that most researchers take for granted that social are unique to human societies because they assume that norm recognition and inference require certain cognitive capacities that only humans share. Given the little agreement about the cognitive architecture that underpins social norms in humans, Andrews *et al*. propose to draw inspiration from the animal culture research program and offer an account of social norms as simple normative regularities. The authors present some cases of potential social norms in animal cultures and discuss what evidence would be needed to judge them as normative.

Kuang & Bicchieri [22] focus on how normative expressions affect norm perception and compliance. They provide evidence that compliance is sensitive to the types of normative expressions and how they are used. In particular, the authors find that people are more likely to comply when the message is framed as an injunction rather than as what most people consider good behaviour. Their results also reveal that behaviour is influenced by the type of normative expression especially when the norm is weak, but not so when the norm is strong.

Finally, Syropoulos *et al.* [23] show how social norms are inferred from public policies. They argue that norms are not only inferred from peers or summary statistics, but that institutional signals, such as the setting of defaults, national laws or policies, can act as coordination devices, signalling or prescribing social norms to large audiences. In two experiments run in the USA, they find that Americans who were randomly assigned to a treatment that informed about their state passing a 100% renewable energy mandate believe that a greater percentage of their state's residents support such a mandate and that the influence of policy signals on social norm perceptions is moderated by information about whether the government represents the will of the people.

### (b) Drivers and consequences of spontaneous norm change

Section 2 of this Theme Issue addresses how, when, and why social norms change spontaneously. Some take an evolutionary perspective and others use the notion of sudden shifts after a tipping point is reached. All contributions present examples of norm change without external (norm-based) interventions and describe how different cultural histories, processes of social learning and environmental contexts brought about processes of norm change. This section starts with a review article on norm formation and change for AI from a complex systems perspective [24]. It proceeds with four research articles that study the emergence and/or consequences of norm change in different domains using survey research [25], agent-based simulations [26], historical data [27], and ethnographic records [28].

Baronchelli [24] in his opinion piece argues that it is crucial to regulate AI and explores three ways in which new social norms might form around the use of AI. He distinguishes different scenarios based on whether the new norm is imposed by a formal authority, by an informal authority, or rather emerges spontaneously in a bottom-up way. On the latter point, the paper reports a conversation with ChatGPT in which the large language model discusses some of the emerging norms it has observed. Baronchelli finally discusses how AI could influence the formation of future social norms and may potentially exacerbate the polarization observed in online social media.

Macanovic *et al.* [26] present social norms as signals that enable group members to distinguish between ingroup ‘friends’ and outgroup ‘foes’, thus facilitating parochial cooperation. They design an agent-based model based on a trust game with signalling to study the dynamics of signalling norm emergence in groups with conflicting interests. They find that minority groups, which benefit from parochial cooperation, develop costly signalling norms from random acts of signalling. Majority groups are less likely to develop costly norms. Only social norms that prescribe sending costless group identity signals can easily emerge in groups of all sizes. Their findings give insight into processes of norm evolutions in contexts where the interests of different groups are not aligned, such as in zones of ethnic conflict or during contests of existing power relations.

Lowes & Nunn [27] take a historical approach and investigate how exposure to slave trades affected kinship structure. By combining historical data on an ethnic group's exposure to the slave trades and the presence of matrilineal kinship following the end of the trades, they show that exposure to the slave trades from 1400 to 1900 CE is associated with matrilineal kinship as observed in the late nineteenth and early twentieth centuries. Their findings contribute to the understanding of the determinants and dynamics of culture and norms.

Globalization processes, to some extent, result in cultural convergence and a loss of cultural diversity. In this process, group differences do exist, and cultural values with different spatial scales might spread or be lost at different rates. In their research article, He *et al*. [28] compare the adoption of cultural values, namely speaking Mandarin or Naru (outgroup languages) and wearing jeans, among the Mosuo and Han, two co-residing ethnic groups in rural southwest China. Their findings support the frequency-dependent selection model, suggesting that factors such as age, sex, education, employment, involvement in tourism, intermarriage and connections in kin networks significantly influence the adoption of cultural norms from outside the group.

Finally, Alvarez-Benjumea *et al.* [25] take as a starting point the demographic evolution of the population in the USA. The USA will soon become a country in which whites are no longer the numerically dominant racial group. Using data from a survey measuring Americans' reactions to racially-offensive speech, Alvarez *et al*. examine whether a social norm controlling anti-white prejudice is emerging in the USA. They find a strong norm against anti-white prejudice amongst white Republicans. The authors discuss these results in the context of polarizing norms.

### (c) Engineering norm change for behavioural change

The final section of the issue discusses how norm change can be engineered. Contributions consider both bottom-up norm enforcement (peer punishment) and top-down interventions (e.g. through norm-based messages) and focus on general norm enforcement or on interventions to pass tipping points. Contributions examine, for instance, the welfare consequences of engineering norm change and how cross-cultural variation in norm enforcement systems impacts the dynamics of social norm change. Interventions for norm change do not come without limitations and some contributions show how norm-based interventions might have backfiring effects [29,30]. This section starts with a review article on punishment as a bottom-up means of norm enforcement across cultures and societies [31]. It proceeds with four research articles that design and test norm interventions to change behavioural intentions in both online [32] and offline settings and in different cultural contexts [33], for norms and meta norms [29], to assess both individual and welfare consequences [30].

Molho *et al*. [31] tackle the issue of variation in punishment across societies. The authors argue that, to date, empirical evidence from cross-societal research has remained disconnected, and they urge for an interdisciplinary integration and an investigation of both the socio-ecological and cultural sources of cross-societal variation in norm enforcement. Their review shows that some aspects of punishment are present in a large set of diverse societies. At the same time, it reveals that evidence on the role of these socio-ecological and cultural factors remains mixed and fragmented, partly because different studies focus on distinct subsets of variables putatively explaining variation in norm enforcement, while excluding other important variables. They finally discuss how variation in norm enforcement systems may affect the dynamics of social norm change.

Pretus *et al*. [32] focus on the spreading of misinformation online. They test the effect of an identity-based intervention to counter the sharing of misinformation. The intervention aims to promote accuracy by incorporating normative cues into the social media user interface. Recent studies show that the success of accuracy-nudging interventions, which are among the most popular approaches to combating misinformation, is relatively weak, especially when the issue is politically polarized. Pretus *et al*. [32] conduct three experiments in the USA and in the UK and find that exposing individuals to normative accuracy judgements by their in-group (versus general others) reduces the likelihood that they will share inaccurate information about partisan issues by 25% (compared to a control condition).

Liu & Lapinski [33] examine the impact of descriptive and injunctive norm appeals on intentions to prevent food waste in China and the USA. Using data from an experimental study, they test the role of cultural context and group orientation in this process. Results show that with the same message exposure, Chinese participants perceive food waste prevention as more prevalent and socially approved compared to US participants. Normative perceptions interacted with cultural context to influence behavioural intentions, such that both descriptive and injunctive norm perceptions predicted stronger intentions to prevent food waste among Chinese participants compared to Americans. These findings suggest the need for the design and implementation of culturally sensitive and contextually appropriate interventions to leverage normative influences effectively.

Vriens *et al*. [29] used the pandemic to track the evolution and change of social distancing norms and meta norms in response to changes in COVID-19 risk. From June 2021 to February 2022, they used a repeated cross-sectional survey design to measure social expectations about social distancing and about the punishment of violations of the distancing norm as the COVID-19 risk first decreased and then increased again. They found that norms and meta norms partially coevolve with risk dynamics, although they recover with some delay. This puts some contraints on their effectiveness to guide preventive behavior against pandemic risk. Comparing behavioral intentions in response to different hypothetical norms and meta norms, their results suggest that the willingness to sanction norm violations increases with a clear meta norm of sanctioning, yet decreases with a clear social norm of distance. Therefore, they conclude with the warning that while standard norm nudges may indeed increase compliance, they may generate negative externalities if strengthening the social norm increases tolerance for norm violations (i.e., because people are less inclined to sanction violations).

Finally, Efferson *et al*. [30] develop two theoretical models that predict when interventions that engineer norm change may be successful and when instead they would backfire. Based on the notion of tipping points, they argue that conformity and coordination can reinforce a harmful social norm, but they can also accelerate change from a harmful norm to a beneficial alternative. While the notion of social tipping is relatively straightforward in homogeneous populations, they show how in heterogeneous populations the effectiveness of an intervention depends crucially on which segment of the population is targeted. They provide theoretical evidence for the counterintuitive finding that an intervention strategy that a tipping intervention that targets the group that is most likely to change their behavior is likely the least effective from a welfare perspective. Instead, an intervention that creates persistent miscoordination—exactly the opposite of tipping—can lead to higher social welfare in the long run.

## 3. Concluding remarks

Interdisciplinary interest has allowed social norms research to advance significantly in the last years, but also produced incongruous definitions and approaches. Moreover, while several significant advances have been made in the study of norm change, developments in one discipline risk being overlooked in another. This makes it increasingly difficult to get a grip on the state of the art in social norms research. This Theme Issue transcends disciplinary boundaries with the aim to offer an integrated theoretical and methodological perspective on norm change that we hope can serve as a reference for past, current and future research on social norms. A cross-article comparison between methods and perspectives lays the foundation for a collaborative, systematic and interdisciplinary research program on social norm change. At a practical level, it presents applications of various methods to measure social norms, test their causal effect on behaviour, and track their evolution and change over time. Lastly, this integrated approach helps to identify novel interventions to promote sustainable cooperation in a variety of urgent collective action problems that our contemporary societies face, like climate change, antibiotic resistance and pandemics, while highlighting also how seemingly straightforward interventions may backfire.

## Data Availability

This article has no additional data.
